# Exploring Consumer Understanding and Perceptions of Front-of-Pack Labelling of Foods and Non-Alcoholic Beverages in Kenya

**DOI:** 10.3390/nu16223892

**Published:** 2024-11-14

**Authors:** Caliph Kirui, Gershim Asiki, Veronica Ojiambo, Caroline H. Karugu, Shukri F. Mohamed

**Affiliations:** 1 Chronic Disease Management Unit, African Population and Health Research Centre, Nairobi P.O. Box 10787-00100, Kenyackarugu@aphrc.org (C.H.K.); smohamed@aphrc.org (S.F.M.); 2 Department of Women’s and Children’s Health, Karolinska Institute, 17177 Stockholm, Sweden; 3 Department of Public and Occupational Health, Amsterdam Medical Centre, Meibergdreef 9, 1105 AZ Amsterdam, The Netherlands

**Keywords:** front-of-pack labels, consumers, perceptions, understanding, non-communicable diseases, Kenya

## Abstract

**Background**: Front-of-package labeling (FOPL) is shown to support healthier consumer choices. Many countries have adopted different FOPL systems. Objective: This study explored perceptions and understanding of three FOPLs and identified features that could enhance their effectiveness in Kenya. **Methods:** A qualitative study was conducted across four Kenyan counties—Nairobi, Mombasa, Garissa, and Kisumu. Data from 12 focus group discussions with 137 adults of diverse socio-demographic backgrounds were analysed. Participants evaluated three FOPLs: Red and Green (RG) Octagon, Red and Green Octagon with icons and text (RGI), and Black Octagon Warning Label (WL). The FGDs assessed visibility and memorability, comprehension, potential effectiveness, and cultural relevance of each label. NVivo version 14.0 was used for thematic analysis. **Results**: Kenyan consumers had mixed perceptions of the proposed FOPLs. The black Octagon WL was found to be the most visible and memorable due to its bright colours. Although the RG and RGI symbols were visually engaging, some participants reported confusion with the colour meanings. The WL was also more readily understood due to its text. Overall, WL was preferred for its potential to influence purchasing decisions, while all three FOPLs were considered culturally suitable. **Conclusions**: The Black Octagon Warning Label was the most visible and comprehensible of the three FOPLs and shows promise in influencing consumer behaviour in Kenya. While RG and RGI symbols are appealing, their colour scheme could reduce their effectiveness. Educating consumers on FOPLs could enhance their impact in reducing unhealthy food purchases.

## 1. Introduction

Non-communicable diseases (NCDs), including heart disease, stroke, diabetes, cancer, and chronic lung disease, are among the leading causes of mortality worldwide, as well as in Kenya. Recent statistics indicate that over three-quarters of all NCD deaths occur in low- and middle-income countries [1]. A significant number of NCDs are attributed to unhealthy diets, which are one of the modifiable risk factors for these diseases [2]. The global increase in overweight/obesity and other nutrition-related non-communicable diseases (NR-NCDs) is associated with the increased availability and overconsumption of unhealthy foods such as ultra-processed foods [3].

To address the global nutrition-related non-communicable disease (NR-NCD) epidemic, the World Health Organization (WHO) has recommended the implementation of effective front-of-pack labelling (FOPL) systems that present clear nutrition information on the front of packaged foods and beverages [4]. These labels guide consumers toward informed food purchases and healthier consumption decisions [5]. There are various types of front-of-pack labels (FOPLs), including traffic light labels, health star ratings, warning labels, and nutrient-specific information. Traffic light labels use color-coding to indicate the healthiness of a product, while health star ratings provide an overall health rating [6]. According to the United Nations Children Fund [7], warning labels highlight specific health risks, and nutrient-specific labels provide detailed information on individual nutrients like sugar, fat, and salt. In Africa, there has been a growing trend towards improving public health through enhanced nutrition information food labels [8]. For instance, South Africa [9] and Ghana [10] have proposed FOPL systems that use warning labels to highlight nutrients exceeding the set thresholds. Although Morocco has not implemented a comprehensive FOPL system, there is increasing emphasis on clear labelling to help consumers in making healthier choices [11]. In Latin America, several LMICs have adopted robust FOPLs to promote healthy eating habits [12]. Chile, for example, has implemented one of the most comprehensive systems, using black warning labels to show foods high in salt, saturated fat, and sugar. Argentina, Mexico, and Peru have also introduced FOPLs similar to Chile’s, while Brazil uses a traffic light system to indicate the nutritional quality of foods [13,14,15].

The effectiveness and comprehension of these labels can vary significantly depending on consumers’ literacy levels and socio-economic status (SES). For example, individuals with lower literacy levels or from lower SES backgrounds may find simple, color-coded, and interpretive systems easier to understand compared to detailed reductive systems [16]. Understanding how different demographic groups perceive and interpret these labels is important before implementing FOPL systems. Studies have shown that perceptions of nutrition labels found on the side, back, or front of food packages vary considerably across different population subgroups [17]. By understanding how various groups interpret FOPLs, decision-makers can design labels that are more effective and equitable.

In response to the rising burden of diet-related NR-NCDs, the Kenyan Ministry of Health (MoH) is considering a FOPL system for packaged foods and drinks. While the Food, Drugs, and Chemical Substances Act has provisions for developing regulations for implementing food labelling measures, including FOPL, no legislation on the same has been developed. Most manufacturers are, therefore, using back-of-pack information, which does not effectively guide consumer purchase intentions. To support the development of effective and equitable FOPL policies in Kenya and generate context-relevant scientific evidence, we aimed to explore consumers’ perceptions of three different FOPL formats.

## 2. Materials and Methods

### 2.1. Study Design and Setting

We conducted a qualitative study using focus group discussions (FGDs). Table 1 presents the distribution of FGD participants by region, socio-economic status, and residence. This study was conducted in four counties in Kenya: Nairobi, Kisumu, Mombasa, and Garissa. The four counties were selected to represent different ethnolinguistic groups, socio-economic contexts, varying access to supermarkets, and diverse consumer behaviours related to packaged food consumption. The recruitment sites were supermarkets located in designated sub-counties, within both low- and mid-high-socioeconomic-status (SES) neighborhoods, as determined by the Kenya National Bureau of Statistics. The sub-counties included in the study were Langata and Embakasi in Nairobi, Kisumu Central and Nyando in Kisumu, Mvita and Kisauni in Mombasa and Garissa Township, and Fafi in Garissa. Data collection took place in October 2023. The methods used in this study adhere to the Consolidated Criteria for Reporting Qualitative Research (COREQ) [18].

### 2.2. Participants and Eligibility Criteria

Twelve [12] FGDs involving a total of 137 adult participants from four counties with a fairly equal representation from both low- and middle-to-high socio-economic statuses were conducted (Table 1). Trained research assistants recruited shoppers as they exited supermarkets. Eligible participants were individuals who frequently purchased packaged foods or drinks and held a primary role in household food purchasing decisions or shared this responsibility within their households. Participants were excluded if they identified as healthcare professionals, employees of the tobacco industry, workers in the sugary drinks and food industry, professionals in advertising, or employees of market research companies.

To capture diverse perceptions across varying socio-demographic groups, we ensured that each quota was adequately represented within each discussion group. This included considerations for gender (male or female), age (18–29, or 30–50 years), education level (no primary school, secondary school, university, and post-graduate school.), income bracket (low or mid-high), and residence (urban and rural) [9]. After receiving sufficient interest from potential participants, we organized convenient venues and times for the FGDs, which were communicated to the participants in advance. Each focus group was homogenous, comprising participants with similar sociodemographic characteristics to facilitate open and relatable discussions. A range of 8–12 participants per group were invited to participate in the focus group discussions.

### 2.3. Study Tools

We used a comprehensive FGD guide (Appendix A) to collect data from the participants. The FGD guide had two sections. The first section of questions explored participants’ perceptions of the symbol’s visibility, memorability, their understanding of Front-of-Pack Labels (FOPLs), the perceived impact on purchasing habits, and their cultural appropriateness. In the second section, participants were asked to identify the FOPL format that was most memorable and impactful to them. The FGD guide was adapted from a similar study conducted in South Africa, ensuring relevance and consistency in data-collection methods [11]. To accommodate participants’ language preferences, the research team translated the FGD into Swahili and Somali. Moderators facilitated discussions in the preferred language of the participants.

### 2.4. Piloting

We conducted two pilot face-to-face FGDs to assess the feasibility of the FGD guide and the overall procedures for the group discussion. Based on feedback from participants and moderators during the pilot discussions, we adjusted the wording and sequencing of questions in the FGD guide.

### 2.5. FOPL Prototypes Tested

**Figure 1:**
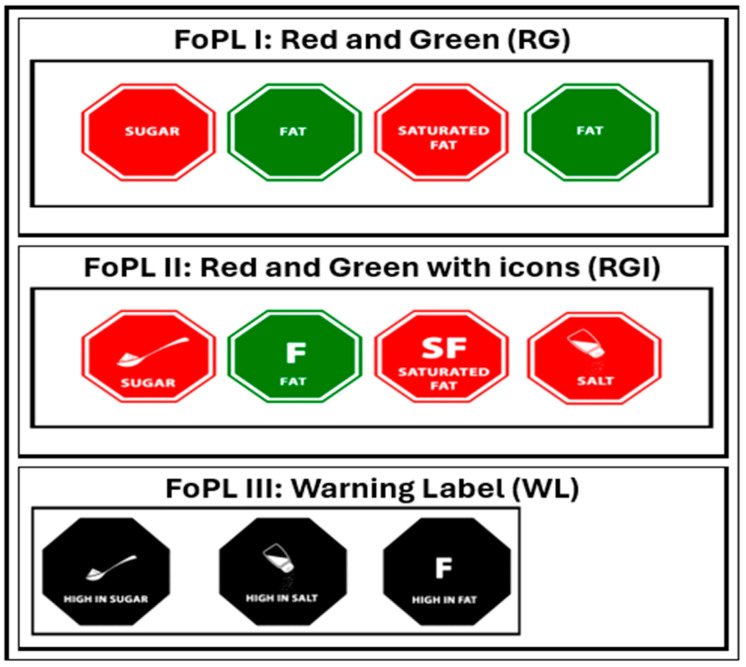
Three front-of-pack labels tested.

FOPL prototypes for testing among Kenyan consumers were carefully selected based on a literature review and recommendations from the Ministry of Health Technical Committee on front-of-pack labelling. The committee identified three symbols (Figure 1) for pre-testing: Red and Green (RG) Octagon, Red and Green Octagon with icons (RGI), and a Black Octagon Warning Label (WL). These symbols were designed to effectively identify nutrients of concern in food products and to promote healthier food choices using color, shapes, text, and icons. A designer was briefed to create visually impactful symbols that would resonate with Kenyan consumers across diverse sociodemographic groups. The goal was to ensure that these FOPLs would be easily understood and effective among diverse groups of the Kenyan population.

### 2.6. FOPLs Tested

Figure 1 shows the three FOPLs tested. The selected FOPL prototypes share common design elements aimed at improving consumer understanding of nutritional information on packaged foods. Each prototype has a distinctive octagon-shaped symbol format. Two of the prototypes, RG and RGI, employ a color-coded system using red to indicate nutrient levels above a specified threshold and green for lower levels as proposed by the Kenya Nutrient Profile Model (KNPM). The RG symbol has text for key nutrients (e.g., “SALT”, “SUGAR”, “FAT”, “SATURATED FAT”), while the RGI symbol had the same text and accompanying icons to visually represent each nutrient: a saltshaker for salt, a teaspoon with sugar for sugar, a large letter “F” for fat, and “SF” for saturated fat. The WL prototype also uses octagonal symbols, but in black with white text, highlighting nutrients exceeding threshold levels with the addition of “HIGH IN” preceding the nutrient name (e.g., “HIGH IN SUGAR”). WL would only appear on food products with the exceeding nutrients of concern.

### 2.7. FOPL Testing Procedures

**Figure 2:**
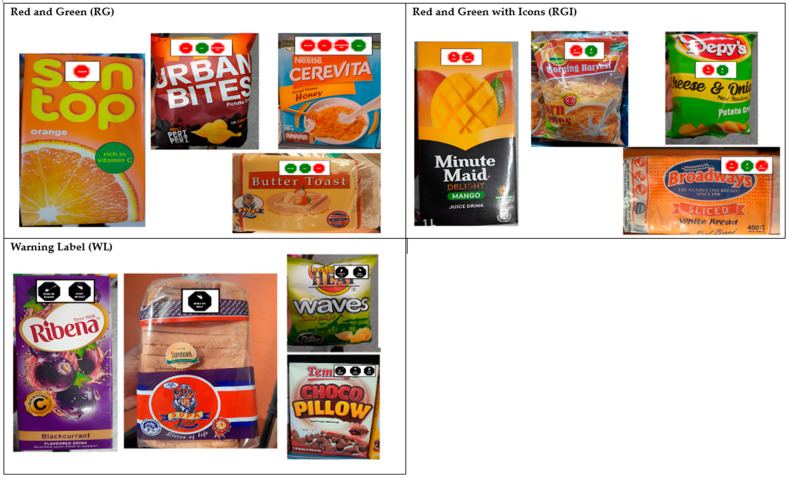
Images used during FGDs.

Images of three FOPL symbols were superimposed onto images of real packages of crisps, juices, breakfast cereals, and breads commonly available in the Kenyan market. The symbols were placed on each package according to their nutrient levels. Four food categories (e.g., breakfast cereals, sugar-sweetened beverages (SSBs), baked goods, and snacks) were selected for testing. In each food category, a set of products with distinct nutrient profiles were developed with varying nutritional quality. Each set of the four products (Figure 2) bearing the respective symbols was printed on laminated A3 cardboard with a clear white background.

### 2.8. Study Procedures

#### 2.8.1. Focus Group Discussions

The FGDs were facilitated by two trained research assistants in designated public facilities such as schools, church buildings, or mosques that were free of noise and interruptions. Each discussion session lasted between 1.5 and 2 hours. To ensure methodological rigor and credibility, the research team conducted sit-ins during all the FGDs and held debriefing sessions with the moderators and research assistants after each session. Participants were reimbursed for their transport costs to and from the venue at the end of the discussion. Written consent was obtained for the FGDs. One research assistant moderated the discussions, while another took brief notes during the sessions. Subsequently, the audio recordings were transcribed verbatim and translated into English for analysis.

#### 2.8.2. Data Collection

Trained moderators facilitated the focus group discussions using an FGD guide prepared by the research team. Each discussion was conducted by one moderator accompanied by a note-taker. At the beginning of each discussion, the moderator handed the first set of images featuring the first FOPL to participants, allowing them to view the images for 10 to 15 s before retrieving them. The moderator then prompted the participants to share what they observed and whether they had noticed any symbols on the products. After gathering their responses, the same images were returned to the participants, who were asked to examine the symbols more closely for another 10 to 15 s. Following this, the images were collected and the moderator initiated a discussion focused on topics such as symbol visibility, memorability, understanding, and perceived impact on purchasing habits. Each aspect was thoroughly discussed until no new insights emerged before moving to the next FOPL. This procedure was repeated for the three FOPLs. Finally, participants were asked to identify the FOPL that stood out the most among the three and provide reasons for their preference.

#### 2.8.3. Ethical Considerations

Ethical approval for the study was obtained from AMREF Ethics and Scientific Review Committee (Approval number ESRC P1323/2022, Approval date 9 March 2023). Study participants were provided with a disclosure page that outlined the study’s purpose and the intent for its subsequent publication. Informed consent was obtained from all participants before conducting the discussions. No direct identifiers were collected, and strict confidentiality measures were maintained throughout the study to ensure participant privacy and confidentiality.

### 2.9. Data Analysis

The data collected were analysed thematically and organized using NVivo version 14.0 following a three-stage process. Initially, transcripts were read multiple times to identify relevant code terms that aligned with the research question. Parent themes were identified, and subsequent readings of the transcripts allowed for the identification of emerging child codes associated with each parent code. Two coders compared their codes and agreed on the codes that best reflected participants’ perceptions. Similar codes were then organized into broader categories and connections between categories were explored. Categories were considered important based on length, depth of discussion, order of emergence, and recurrence across multiple focus groups. The coders harmonized the codebook used to analyse all 12 transcripts from the study. Additionally, a qualitative data-analysis expert reviewed the data both before and during analysis. Thematic analysis was used to identify recurrent themes and patterns within the qualitative data.

## 3. Results

### 3.1. Participant Socio-Demographics

Table 2 shows the socio-demographic characteristics and purchasing responsibilities of participants by county. A total of 137 adult participants took part in the study across 12 focus group discussions. Among the respondents, 71 (51.8%) were male, a slight majority (54.0%) were aged 18–29, and most (75.2%) had education levels above secondary. Over half (58.4%) were parents of children under age 18 years, and 68.6% were the main decision-makers in their households. Nearly half (47.4%) bought their food items from shops and kiosks, 29.9% from supermarkets, and 43.8% purchased packaged foods daily.

### 3.2. Themes

Table 3 provides the themes and sub-themes. Four themes and eleven sub-themes describing participants’ perspectives on front-of-pack symbols were extracted deductively from the data collected. These included [1] visibility and memorability, which assessed how easily participants could see and recall the labels; (2) comprehensibility, which evaluated participants’ understanding of the information conveyed by the symbols’ (3) potential effectiveness, which examined participants’ perceptions of the labels’ influence on their purchasing decisions, and (4) cultural appropriateness, which explored the cultural relevance and acceptability of the symbols. Each theme provided a structured approach to gather valuable insights into the potential impact and effectiveness of the three FOPLs tested.

### 3.3. Visibility and Memorability

Participants’ visibility and memorability were explored through the labels’ colour, shape, and text. The RG Octagon symbol was considered attractive and visible, while some participants found it memorable due to its simplicity. This was mainly attributed to the use of bright colours, which grabbed the participant’s attention quickly. The RGI Octagon symbol was also found to be quite visible and generally deemed memorable. However, for both the RG Octagon and RGI Octagon symbols, it emerged that the text used was found to be unclear to many participants.

*“The label is attractive because of the eye-catching color.”*

*[Label RG, female…R10, FGD3, Langata, Nairobi County].*

*“It was visible, and I think they added pictures on top of the writings, I took note of that.”*

*[Label RGI, male…R8, FGD3, Mvita, Mombasa County].*

The WL Octagon symbol was viewed as the most visually appealing and memorable. While study participant preferences differed, the WL Octagon symbol, with its black and white colours and informative text, emerged as the most consistently memorable among most study participants.

*“Okay, I can say the most attractive thing in that last label [WL], ummm, it’s that color, it matches, black and white”*

*[Label WL, male…R2, FGD2, Kisauni, Mombasa County].*

*“The label’s colour caught my attention, and its black colour was highly visible.”*

*[Label WL, male…R1, FGD1, Garissa Township, Garissa County].*

*“The text information in the label [WL]’High sugar and High salt’ enhances its memorability…”*

*[Label WL, male…R2, FGD1, Garissa Township, Garissa County].*

### 3.4. Comprehensibility

Participants provided diverse perspectives on how easily the three front-of-pack symbols were understood. While some found RG and RGI symbols easy to understand, associating red with danger, others expressed challenges in interpreting the labels due to a lack of clear indications of salt and sugar quantities. Other participants expressed that the purpose of the colour-coded symbols was to indicate the content of the items in the packaged product. Some of the participants were able to understand that the use of red colour was in a way associated with danger.

*“I know red means danger, so I was able to distinguish that this stuff is not good, because of the color, because it is in red”*

*[Label RG, male…R5, FGD1, Kisauni, Mombasa County].*

*“It does not indicate the ratio of salt and sugar. Thus, it’s not easy for one to tell whether it has too much sugar or salt”*

*[Label RG, female…R3, FGD1, Embakasi, Nairobi County].*

*“It was easy to understand because the ingredients have been quantified”*

*[Label WL, female…R10, FGD1, Nyando, Kisumu County].*

However, concerns were raised about potential confusion when the same colours (red and green) were used to represent different contents. For both the RG Octagon and RGI Octagon symbols, it emerged that the text used was found to be unclear to many participants.

*“The labels [RG] are memorable if they are few. For example, there was a brand which had few labels namely sugar and salt alone. … if there are more than four contents, I would not remember”*

*[Label RG, female…R8, FGD2, Embakasi, Nairobi County].*

We also found that there was barely any mention of the green label in the colour-coded labels. However, some participants thought it represented sugar, while a few thought it indicated that the product is good for consumption. Despite varying opinions, WL was frequently mentioned as the most understood as it provided additional informative text.

*“For example, if a product has that, green package for sugar it tells us that there is sugar content in it, the only thing is it doesn’t tell us what level it is.”*

*[Label RG, male…R6, FGD2, Langata, Nairobi County].*

*“The label shows that the product is good for consumption because it contains fat and sugar which are green in colour.”*

*[Label RG, female…R2, FGD1, Embakasi, Nairobi County].*

Many participants felt that the warning labels aimed to deter them from consuming these products in excess. When asked who they thought the labels were designed to communicate with, participants felt that the warning labels were meant for people who were obese and wanted to lose weight, those with high blood pressure who were advised to reduce salt intake, and those with diabetes who needed to avoid sugar.

*“It warns the consumers, if you don’t use high level sugar, it warns you not to consume”*

*[Label WL, female…R10, FGD2, Kisumu Central, Kisumu County].*

*“…it is warning me about the content that is high…”*

*[Label WL, male…R7, FGD3, Langata, Nairobi County].*

Participants generally believed that Kenya’s nutrition-related non-communicable diseases (NR-NCDs) could be effectively addressed by using the three labels. However, many felt that the WL symbol might have the most significant impact due to its comprehensive information. Most participants preferred the WL symbol over the other two colour-coded labels, highlighting the WL’s potential to influence healthier dietary choices.

Certain aspects of the coloured labels were difficult for participants to understand. The use of different colours, such as RG and RGI labels, in relation to various contents like sugar and salt, was particularly confusing. Participants expressed difficulty in grasping the rationale behind the varying colours for different contents, leading to uncertainty about what each colour specifically indicated. This confusion was compounded when multiple colours were used on a single package, making it harder for participants to quickly and accurately interpret the labels’ messages.

*“Why do they use red color on both salt and sugar labels?”*

*[Label RGI, female…10, FGD3, Langata, Nairobi County].*

*“The colours of the label are so confusing thus leading to a lack of understanding of product labels”*

*[Label RG, male…R1, FGD2, Garissa Township, Garissa County].*

Participants highlighted several key points regarding the symbol message. They noted that the primary concept of the colour-coded labels was generally understood. For instance, the colour red was commonly associated with danger. However, most participants found the messages in the warning labels to be clearer and more direct, particularly when text was included.

*“I know red means danger, so I was able to distinguish that this stuff is not good, because of the color, because it is in red.”*

*[Label RG, female…R5, FGD2, Kisauni, Mombasa County].*

*“Because in this label, there is an indication telling us that this product has high sugar, high fat, high salt, while in the other labels, it was just saying salt, sugar or fat. It was not telling us whether it is high or low…”*

*[Label WL, male…R6, FGD1, Nyando, Kisumu County].*

### 3.5. Potential Effectiveness

Participants generally reported that their purchase decisions would change if they saw these labels on packaged products. Many indicated they would be more likely to choose healthier options, especially if a warning label was present. Some participants described intentions to reduce consumption of products high in sugar and salt. Overall, the majority felt that warning labels would guide them towards healthier food choices.

*“I think that when I go to buy, I will be 50–50, because this label, you know, its red, so it is danger.”*

*[Label RG, male…R3, FGD2, Embakasi, Nairobi County].*

*“Yes, the label [WL] has the potential to change my attitude towards the products …”*

*[Label WL, female…R1, FGD3, Fafi, Garissa County].*

*“… If the products will be well labelled, it will make the consumer to make a very informed decision of what not to eat. So, it will enable us to stay healthy. So, it will help avoid some of the lifestyle diseases. Things like obesity…”*

*[Label RG, female…R2, FGD1, Kisauni, Mombasa County].*

### 3.6. Cultural Appropriateness

While some participants considered the labels essential for identifying daily sustenance in their culture, others pointed out that the colour red might be a turn-off, while the black colour is thought to have significance in funeral attire. Overall, opinions were mixed, but most respondents perceived the symbols as culturally appropriate, though a few others advocated for adjustments to better resonate with cultural perceptions.

*“We believe that anything red means danger. But it doesn’t affect culture, it only promotes it.”*

*[Label RGI, male…R8, FGD3, Nyando, Kisumu County].*

*“We always believe black is funeral attires”*

*[Label WL, female…R6, FGD2, Kisumu Central, Kisumu County].*

*“This label has nothing to do with our culture… it is okay”*

*[Label RG, female…R4, FGD1, Mvita, Mombasa County].*

## 4. Discussion

This study explored Kenyan consumers’ perceptions on three front-of-pack labels (FOPLs): RG, RGI and WL, focusing on their visibility, memorability, comprehensibility, potential effectiveness, and cultural appropriateness. The findings revealed diverse perceptions among the study participants, with a notable preference for the WL due to its clarity and comprehensive information.

Participants found the RG label visually appealing due to the bright colours, and that red is associated with danger. Participants were generally silent about the green colour in the labels where it was present. Additionally, there was confusion when the same colours were used to represent different nutrients such as salt, sugar, or fat. Their varied views of the three labels suggest that visual appeal, colour contrast, and simplicity and clarity of text significantly impact how well these labels are noticed and remembered. The coloured labels, with their distinct red and green colours, were generally regarded as attractive and easily visible. However, the potential for confusion when the same colours were used for different nutrients points to the complexity of how participants balance visibility with clarity. These findings are supported by Temple and Fraser [19], who stressed the importance of consistent and insightful labelling to avoid consumer misinterpretation. The warning label (WL) was perceived as the most visually appealing and memorable. This pattern was also noted in findings from a previous study in South Africa, where participants appreciated that warning labels were readily visible on the pack with many observing that the black colour makes it easily noticeable [9].

The observed preference for the WL among most of the study participants can, therefore, be attributed to its simplicity associated with a monochromatic colour, informative text “High in…”, and the simple pictorials. Similar findings were reported in a previous study, which found that the number of nutrients that consumers prefer on the front of the pack was often four or less [20]. These results offer insightful information for creating memorable and visually appealing front-of-pack symbols that can support consumers in making well-informed product-selection decisions. They also highlight the significance of visual elements like contrast and colour selection in improving both the visibility and recall of front-of-pack symbols. The development and application of front-of-pack symbols to efficiently convey nutritional information to consumers while enhancing their visibility and memorability can, therefore, be informed by these observations.

The varying levels of comprehensibility among the three front-of-pack labels (FOPL) labels used in the study highlight the importance of clear and accessible nutrition labelling, particularly in the context of low- and middle-income countries like Kenya. The preference for the WL observed among participants underscores the effectiveness of explicit and straightforward messaging in nutrition communication. These findings align with existing literature, which suggests that warning labels with clear text can significantly enhance consumer understanding, particularly when compared to more complex labelling systems that rely solely on colour coding [21]. As seen from the results, it was clear that when you have food with varied nutrients, consumers would seriously confuse the colours. They tended to think colours stand for the various nutrients. Besides this, there is no clear interpretation of the green colour among the participants. This was evident when some participants associated red colour with one nutrient and green with another, e.g red representing sugar and green representing fat. The challenges in comprehension that participants faced with the colour-coded labels, particularly the confusion around the use of multiple colours for different nutrients, point to potential limitations of such a system when not carefully designed or contextualized [22,23].

South Africa’s ongoing endeavors to implement WLs highlight an emerging interest among African countries to promote public health policies that prioritize consumer well-being [9]. Our findings also reflect recent research evidence that has shown that interpretive FOPL systems are preferred in LMICs owing to low consumer nutrition literacy [24]. The simplicity and clarity of the WLs, as preferred by the participants, align with evidence suggesting that consumers are more likely to accept warnings that are straightforward and easily understood [25]. One of the key factors contributing to enhancing participants’ understanding was the inclusion of additional informative text. In a study conducted in China, most participants preferred the black warning label shield, which showed in text the high content of the nutrients of concern. This preference demonstrated that such WLs were much easier to understand and offered a simpler method of choosing healthier food options [25]. A review of FOPLs noted the need for clear colour and message congruence. The review suggested that if a colour-based system is to be used, it should be set against a black and white background to ensure it stands out from the packaging [26]. The results, therefore, highlight the importance of including colour and text as interpretation aids in new FOPLs as reported by Pettigrew [27].

The potential for the WL to discourage purchase and consumption of unhealthy foods was evident from our findings. Participants indicated a strong likelihood of modifying their purchase decisions in favour of healthier options when they saw clear and direct labelling, particularly the warning labels (WLs). This finding is consistent with previous studies, such as those by Temple and Fraser [19], which showed that warning labels significantly influence consumer behaviour by providing immediate cues about the health risks associated with certain food products. Additionally, the participants’ belief that FOPLs could help prevent lifestyle-related diseases highlights the broader public health implications of effective labelling strategies. Research by Jones et al. [28] supports this view, indicating that well-designed FOPLs can lead to sustained changes in consumer behaviour, thereby contributing to the prevention of NCDs at a population level. Similarly, a recent study in South Africa found that most participants anticipated WLs would reduce their purchase of unhealthy foods [9]. In Brazil, de Morais Sato et al. [29] found that most women believed that WLs would reduce their consumption of unhealthy foods for both them and their children, while men were divided between stopping consumption entirely or continuing without altering their intake. These findings, therefore, highlight the potential for the WLs to influence purchase and consumption behaviours of unhealthy foods.

Cultural appropriateness is an important consideration in the design and implementation of front-of-pack labels (FOPLs), particularly in diverse societies like Kenya, which has at least three ethnic groups (Bantu, Nilotes, and Cushites) and more than 40 languages [30]. Although all participants did not find the labels explicitly culturally inappropriate, some raised concerns about the use of the colour red, which they associated with danger. This suggests that cultural perceptions of colour can influence consumer reactions to food labels. These findings align with findings from a study by Becker et al. [31], who noted that cultural symbols and colour associations can significantly impact how nutritional information is interpreted. To ensure broader acceptance and effectiveness, neutral colours may be more appropriate for FOPLs to better align with cultural norms and values. Finally, when developing FOPL policies, governments should follow the available evidence. A substantial evidence base exists on the most effective FOPLs [26], and the WHO offers guidance to help identify and implement the most appropriate and effective labeling system for national contexts [32].

### Strengths and Limitations

One of the primary strengths of this study is its focus on exploring consumer perceptions of front-of-pack labels (FOPLs) in Kenya, where context-specific evidence on nutrition labelling is limited. This makes it the first of its kind in East Africa to explore perceptions on FOPLs, contributing to its novelty. The qualitative nature of the study allowed for in-depth discussions, capturing diverse and nuanced perspectives that are often missed in quantitative methods. Additionally, the inclusion of participants from four counties with varying socio-economic statuses enhances to the generalizability of the findings within the Kenyan context. By focusing on three different FOPL formats, the study offers a comprehensive understanding of how different labelling systems are perceived, allowing for a comparison of their potential effectiveness. Despite these strengths, the study did not explore long-term changes in behaviour, which means the potential impact of these labels on actual purchase decisions and health outcomes remains uncertain.

## 5. Conclusions

This study provides valuable insights into consumer perceptions of front-of-pack labels in Kenya, highlighting both the potential and challenges of using these labels to influence healthier food choices. The findings show that warning labels (WL) were generally the most preferred and easily understood among Kenyan consumers. The study underscores the importance of considering local contexts and cultural factors into account when designing and implementing FOPL systems. As Kenya moves toward adopting such labelling policies, the Ministry of Health should consider using the Black Octagon warning label to effectively guide purchase and consumption behaviours while contributing to the reduction of diet-related non-communicable diseases.

## Figures and Tables

**Table 1:** Distribution of FGD participants by region, socio-economic status (SES) and urban–rural location.

County	Location	Focus Group Discussions (FGD)	Number of Participants	SES	Urban–Rural Location
Nairobi	Langata	FGD 1	11	Mid-high	Urban
	Embakasi 1	FGD 2	11	Low	Urban
	Embakasi 2	FGD 3	12	Low	Urban
Mombasa	Mvita	FGD 1	10	Mid-high	Urban
	Kisauni 1	FGD 2	10	Low	Rural
	Kisauni 2	FGD 3	12	Low	Rural
Kisumu	Kisumu Central 1	FGD 1	10	Mid-high	Urban
	Kisumu Central 2	FGD 2	12	Mid-high	Urban
	Nyando	FGD 3	12	Low	Rural
Garissa	Garissa Township	FGD 1	11	Mid-high	Urban
	Garissa Township 2	FGD 2	9	Mid-high	Urban
	Fafi	FGD 3	15	Low	Rural

**Table 2:** Socio-demographic characteristics of study participants by county.

Variables	Nairobi (*n* = 34)	Kisumu (*n* = 36)	Mombasa (*n* = 32)	Garissa (*n* = 35)	Total (*N* = 137)
**Sex**					
Male	17 (50.0%)	18 (50.0%)	16 (50.0%)	20 (57.1%)	71 (51.8%)
Female	17 (50.0%)	18 (50.0%)	16 (50.0%)	15 (42.9%)	66 (48.2%)
**Age category**					
18–29 years	17 (50.0%)	16 (44.4%)	19 (59.4%)	22 (62.9%)	74 (54.0%)
30–50 years	17 (50.0%)	20 (55.6%)	13 (40.6%)	13 (37.1%)	63 (46.0%)
**Education level**					
No Primary school	0 (0.0%)	0 (0.0%)	0 (0.0%)	5 (14.3%)	5 (3.6%)
Primary school	8 (23.5%)	12 (33.3%)	2 (6.2%)	4 (11.4%)	26 (19.0%)
Secondary	12 (35.3%)	10 (27.8%)	6 (18.8%)	18 (51.4%)	46 (33.6%)
College/University	14 (41.2%)	13 (36.1%)	23 (71.9%)	7 (20.0%)	57 (41.6%)
Postgraduate school	0 (0.0%)	1 (2.8%)	1 (3.1%)	1 (2.9%)	3 (2.2%)
**Parent or caregiver for children aged below 18?**					
No	6 (17.6%)	4 (11.1%)	18 (56.2%)	29 (82.9%)	57 (41.6%)
Yes	28 (82.4%)	32 (88.9%)	14 (43.8%)	6 (17.1%)	80 (58.4%)
**Main decision-maker for food purchases**					
No	9 (26.5%)	3 (8.3%)	9 (28.1%)	22 (62.9%)	43 (31.4%)
Yes	25 (73.5%)	33 (91.7%)	23 (71.9%)	13 (37.1%)	94 (68.6%)
**Main buyer of food**					
Yes	21 (61.8%)	29 (80.6%)	23 (71.9%)	13 (37.1%)	86 (62.8%)
Shared responsibility	13 (38.2%)	7 (19.4%)	6 (18.8%)	12 (34.3%)	38 (27.7%)
Not the main buyer	0 (0.0%)	0 (0.0%)	3 (9.4%)	10 (28.6%)	13 (9.5%)

**Table 3:** Key themes and sub-themes.

Themes	Sub-Themes
1. Visibility and memorability	Colour
	Shape
	Text
	Memorability
2. Comprehensibility	Ease of understanding
	Purpose and audience of symbol
	Clarity and confusion
	Label message
3. Potential effectiveness	Shift on purchase intention
	Effect of symbol on diet related NCDs
4. Cultural appropriateness	Cultural appropriateness

**Figure A1:**
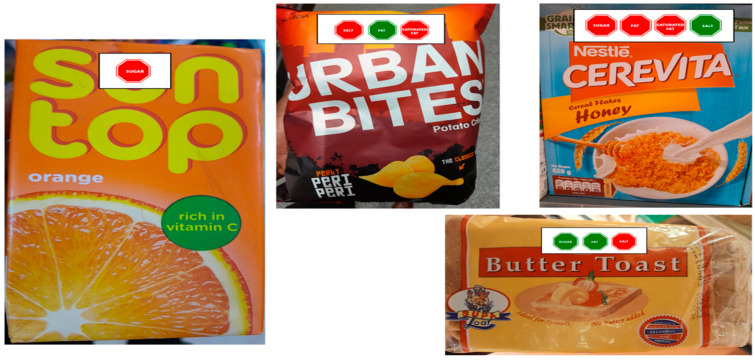
Red and Green Symbol.

**Figure A2:**
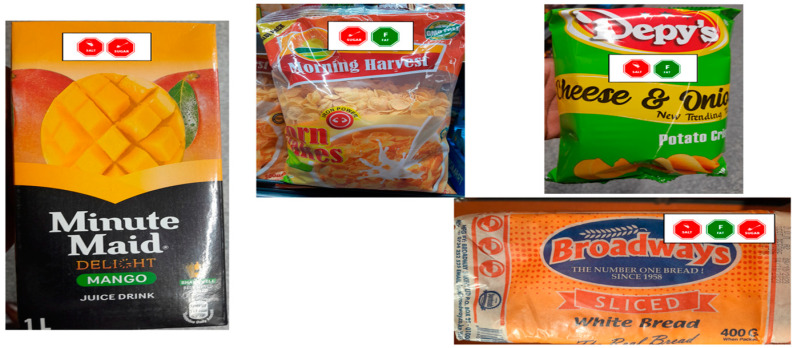
Red and Green symbol with icons.

**Figure A3:**
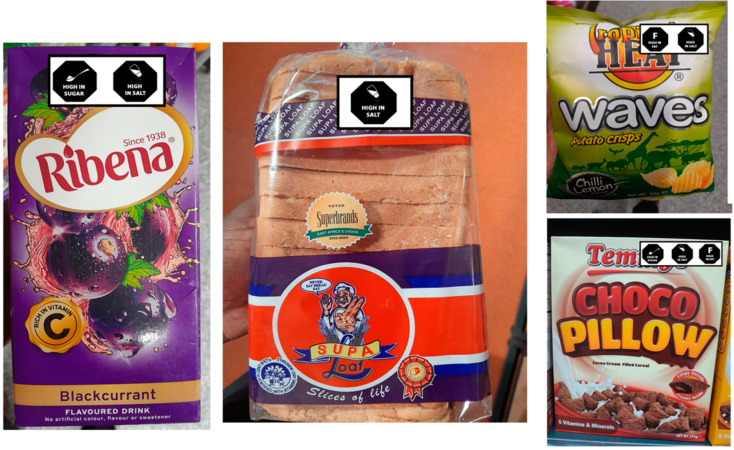
Warning Symbol.

## Data Availability

The data that support the findings of this study are available from the corresponding author upon reasonable request.

## Appendix A. Focus Group Discussion Guide

Introduction

- Shortly introduce yourself (e.g., where do you work, how you are involved in the project).
- Thank stakeholders for participation.
- Explain the research project.
- Explain the aim of the interview.
  - *Interested in exploring participants’ understanding of front-of-pack labels, the label’s perceived effect on purchasing habits, visibility and credibility.*
  - *Explore formats perceived as attention grabbing, effective format to use as a warning against unhealthy foods and drinks, and formats likely to influence purchase behaviour.*
- Explain informed consent and ask stakeholder to sign.
  - *Make sure that the stakeholder has your contact details if any additional questions would come up later.*
  - *Explain that the audio recorder can be paused at any time during the interview.*
  - *Explain that the stakeholder can request for any parts of the audio recordings to be removed after the interview, up to [date].*
  - *Explain that the results will only be described and reported at an aggregated level.*
- Before proceeding, ask if there are any questions about the research project, the interview or the confidentiality.

Ask permission to turn on the recorder.

Opening questions

Hello, my name is ___________, I will be conducting the session today, and this is __________, who will assist me by taking notes and helping in the conduct of the research. Thank you for participating in this study today. We’re interested in people’s reactions to proposed labels for the front of food and drink packages. I will show you a label that is being proposed. I will ask you for your reactions to it. Now, I am going to show you an image of some products and then we will discuss what you see. First, I want to make sure that you can see the image clearly, so I will show you a trial image and once you confirm that you can see it clearly, we will proceed to the main image.

[Figure A1, Figure A2 and Figure A3]

Did you notice any labels on the packages?
Now I am going to show you the image again and this time I want you to focus closely on a set of labels you will see on the front of the food and drink packages.
Study this set of labels closely and l will ask you some questions about it.
*[VISIBILITY/MEMORABILITY]*
How visible was the label?
Describe how eye-catching you found it to be (Probe: shape, color, text, message)
How memorable is the label?
Kindly recall and describe the label to me. (Probe on shape, color, text, message)
*[COMPREHENSIBILITY]*
What do you think is the purpose of these labels?
What did you understand from the labels?
What does the label tell you about the healthiness of the food?
Was it easy to understand? Why?
What did you not understand or find confusing?
To whom do you think this label is directed to?
How believable did you find the label?
What about this label may be culturally inappropriate?
What about this label may be difficult to understand/interpret in Kenya?
*[POTENTIAL EFFECTIVENESS]*
Did this label change your attitude towards the food/drink that you just saw? How?
Would your decision to buy a product in the shop be affected if you saw this label on a product? How?
Would this label affect your purchase of a product that you regularly buy if it was on it? Probe: How, increase or decrease the purchase of it?
In your opinion, what is the benefit of placing a label like this on healthy/unhealthy foods?
How would this label be harmful?
What effect does a label like this have on people in Kenya? Probe—*how will it help in addressing obesity or high blood pressure?*
*[IMPROVEMENTS TO THE LABEL’S MEMORABILITY]*
What aspect of the label had the most impact on you and why?
Was there anything that was offensive or inappropriate in the labels?
*[COMPARATIVE RATING]*
Now, thinking of all the labels you’ve seen today, what was the most memorable in these labels? *Probe—what image do you remember most—color, shape, text? What words do you remember?*
Thinking of all the labels you’ve seen today, does any one label stand out for you? Which one would that be? Why?
